# *M. tuberculosis* CRISPR/Cas proteins are secreted virulence factors that trigger cellular immune responses

**DOI:** 10.1080/21505594.2021.2007621

**Published:** 2021-12-09

**Authors:** Jianjian Jiao, Nan Zheng, Wenjing Wei, Joy Fleming, Xingyun Wang, Zihui Li, Lili Zhang, Yi Liu, Zongde Zhang, Adong Shen, Li Chuanyou, Lijun Bi, Hongtai Zhang

**Affiliations:** a Key Laboratory of RNA Biology and State Key Laboratory of Biomacromolecules, Cas Center of Excellence in Biomacromolecules, Institute of Biophysics, Chinese Academy of Sciences, Beijing, China; b University of Chinese Academy of Sciences, Beijing, China; c Beijing Chest Hospital, Capital Medical University; Beijing Tuberculosis and Thoracic Tumor Research Institute; Beijing Key Laboratory for Drug Resistant Tuberculosis Research, Beijing, China; d Beijing Pediatric Research Institute, Beijing Children’s Hospital, Capital Medical University, Beijing, China

**Keywords:** Secreted virulence factors, innate immunity, Mycobacterium tuberculosis, CRISPR/Cas proteins

## Abstract

The role of prokaryotic CRISPR/Cas system proteins as a defensive shield against invasive nucleic acids has been studied extensively. Non-canonical roles in pathogenesis involving intracellular targeting of certain virulence-associated endogenous mRNA have also been reported for some Type I and Type II CRISPR/Cas proteins, but no such roles have yet been established for Type III system proteins. Here, we demonstrate that *M. tuberculosis* (Type III-A system) CRISPR/Cas proteins Csm1, Csm3, Csm5, Csm6, and Cas6 are secreted and induce host immune responses. Using cell and animal experiments, we show that Cas6, in particular, provokes IFN-γ release from PBMCs from active tuberculosis (TB) patients, and its deletion markedly attenuates virulence in a murine *M. tuberculosis* challenge model. Recombinant MTBCas6 induces apoptosis of macrophages and lung fibroblasts, and interacts with the surface of cells in a caspase and TLR-2 independent manner. Transcriptomic and signal pathway studies using THP-1 macrophages stimulated with MTBCas6 indicated that MTBCas6 upregulates expression of genes associated with the NF-κB pathway leading to higher levels of IL-6, IL-1β, and TNF-α release, cytokines known to activate immune system cells in response to *M. tuberculosis* infection. Our findings suggest that, in addition to their intracellular shielding role, *M. tuberculosis* CRISPR/Cas proteins have non-canonical extracellular roles, functioning like a virulent sword, and activating host immune responses.

## Importance

Tuberculosis, caused by *Mycobacterium tuberculosis*, is an ancient but resurgent disease and has been the leading cause of death from an infectious agent for many years. Poor understanding of the basic biology of this pathogen limits development of much needed new drugs, diagnostic tools and vaccines. The prokaryotic CRISPR/Cas system has long been thought of as an intracellular defense system against foreign nucleic acids, but its potential involvement in bacterial pathogenesis and virulence has not been widely considered. Here we report that this bacteria’s Type III-A CRISPR/Cas system proteins are secreted antigens that provoke an immune response, and that certain of its CRISPR/Cas proteins can induce macrophage apoptosis, induce the innate immunity NF-κB signaling pathway, and provoke the release of cytokines that are known to activate immune system cells in response to *M. tuberculosis* infection. The virulence and pathogenic mechanisms of these CRISPR/Cas proteins merit further investigation.

## Introduction

*Mycobacterium tuberculosis* (MTB), the etiological agent of tuberculosis (TB), is considered to be one of the most successful pathogens and has evolved a range of mechanisms for evading the host immune system [1]. The annual death-toll of this ancient yet resurgent disease was estimated as 1.4 million in 2019 [2], and the impact of the COVID-19 pandemic is expected to lead to increases in TB cases and deaths in coming years [2]. Greater understanding of the range of mechanisms by which MTB evades the host immune system is needed to catalyze development of a much needed new generation of anti-tubercular drugs and vaccines to combat this global threat to public health.

During the early stages of infection, the extent of bacterial survival and proliferation is mainly determined by the efficacy of the host innate immune response, macrophages being the main effector cells [3]. On the one hand, the virulence of MTB depends on its ability to invade, persist, and replicate within macrophages [4]. Infection with MTB, on the other hand, significantly increases rates of induced macrophage apoptosis *in vitro* [5]. Two elements are key in this process, namely the mycobacterial factors that modulate macrophage apoptosis, and the host macrophage apoptosis associated pattern recognition receptors (PRRs), such as Toll-like receptors (TLRs) TLR-2 and TLR-4 [6]. Once PRRs recognize mycobacterial apoptosis-associated factors, they activate various adaptor proteins and release pro-inflammatory cytokines, in turn initiating immune responses to attack pathogens [7]. To date, only a few MTB apoptosis-inducing factors have been identified, the best known of which, ESAT-6 (Rv3875), has been shown to induce THP-1 cell apoptosis [8]. LpqH (Rv3763) and PPE32 (Rv1808) have also been found to trigger apoptosis [9,10].

Like other prokaryotes, MTB has a CRISPR/Cas (Clustered Regularly Interspaced Short Palindromic Repeats and associated genes) adaptive immune system for defense against foreign nucleic acids, its Class I, Type III-A CRISPR/Cas system consisting of an effector complex composed of proteins Csm1-5 and crRNA [11]. Some proteomic studies have included MTB CRISPR/Cas proteins Csm1, 3, 5, and Cas6 on lists of proteins in MTB culture filtrates [12,13] (Supplementary Table 1), providing a hint that in addition to their role in intracellular defense, these proteins may also be involved in host-pathogen interactions, but this has yet to be investigated. Non-canonical roles encompassing gene regulation, stress responses, and bacterial virulence have been reported for some CRISPR/Cas proteins from other systems [14–17]. *Francisella novicida* Cas9, for example, acts together with scaRNA (a non-crRNA) to regulate gene expression via the degradation of an endogenous mRNA, leading to innate immune evasion and promoting virulence [18].

Here, using cell and animal studies, we sought to confirm that MTB CRISPR/Cas proteins are secreted and then investigate any non-canonical roles they play in host–pathogen interactions, particularly with respect to the induction of host immune responses, including macrophage apoptosis. We report that Csm1, Csm2, Csm3 and Csm5, Csm6 and Cas6 are secreted antigens, and that Cas6 in particular plays a role in virulence. Cas6 can induce macrophage apoptosis and it activates the macrophage NF-κΒ signaling pathway, leading to the release of cytokines important in the activation of immune cells. Our study not only sheds light on unexpected extracellular roles of MTB CRISPR/Cas proteins, but also suggests these proteins merit investigation as TB biomarkers and/or vaccine candidates.

## Results

### MTB CRISPR/Cas proteins are secreted and induce a host T-cell immune response

**Figure 1.**
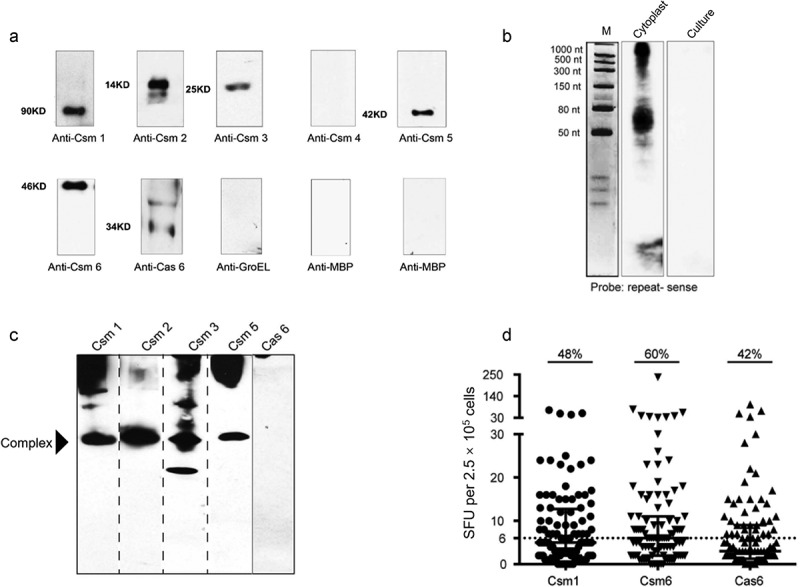
Crispr/Cas Proteins Are Secreted And Induce A Host Immune Response. (a) Western blotting of 30 d MTB H37Rv culture filtrates (50 μg protein) with anti-Csm sera (1 μg/ml). Control: GroEL (unsecreted protein; 50 μg). (b) Northern blotting of culture filtrates with an MTB CRISPR repeat (sense) probe. Control: cytoplasmic RNA. (c) Secreted CRISPR/Cas proteins may form an extracellular complex. Native gel Western blotting of culture filtrates (50 μg protein) with anti-Csm sera (1 μg/ml). (d) Stimulation of PBMCs from active TB patients with recombinant Csm1, Csm6 and Cas6 provokes IFN-γ release. Dot plot showing spot forming units (SFU) per 2.5 × 10^5^ PBMCs, as determined with ELISPOT assays. Horizontal lines: median SFUs per group. SFUs ≥6: positive (dotted line). All experiments in A-C were performed at least three times. Images presented are from representative experiments

To determine if CRISPR/Cas proteins are indeed secreted, we performed western and northern analyses of MTB culture filtrates using denaturing gels Figure 1(a,b); we were able to detect Csm1, Csm2, Csm3, Csm5, Csm6 and Cas6, but not Csm4 or crRNA. When western analysis was performed on MTB culture filtrates using native gels Figure 1(c), bands for Csm1, Csm2, Csm3, and Csm5 were detected on Western blots at almost the same position, suggesting that even in the absence of Csm4 and crRNA, these proteins, like their corresponding intracellular proteins, may form a complex. Cas6 was also detected extracellularly Figure 1(a); Supplementary Figure 1), but was not associated with the putative Csm1, 2, 3, 5 complex Figure 1(c). These results led us to hypothesize that CRISPR/Cas proteins, like other secreted proteins, may be involved in host–pathogen interactions.

As most known MTB-specific T-cell antigens are secreted proteins important in the induction of protective immune responses, and IFN-γ release is necessary for the control of bacterial growth and host survival in both humans and mice [19], we used ELISPOT assays to investigate if representative secreted CRISPR/Cas proteins (Csm1, Csm6 and Cas6, selected randomly) could provoke IFN-γ release from peripheral blood mononuclear cells (PBMCs) from active TB donors Figure 1(d), Supplementary Table 2). All three proteins provoked IFN-γ release in a substantial proportion of active TB donor PBMC samples (48%, 60%, and 42%, respectively, vs 72% and 58%, respectively, for the ESAT-6 and CFP-10 antigen controls) (Supplementary Table 2), demonstrating that they are antigens that induce a host immune response.

### Secreted MTBCas6 is a virulence factor

**Figure 2.**
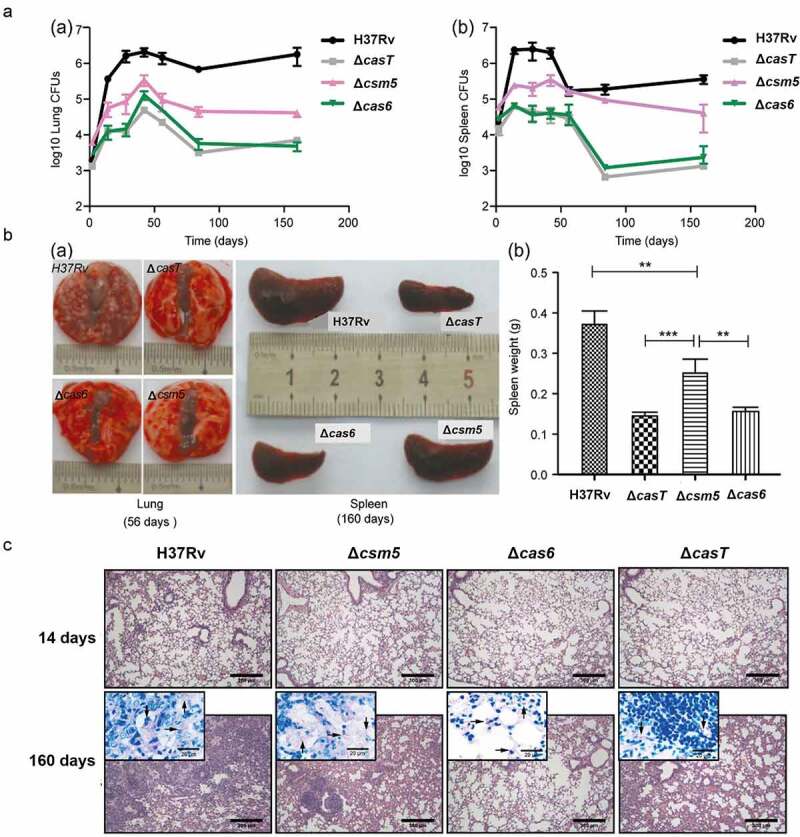
Secreted MTBCas6 is an important virulence factor in MTB pathogenesis. (a) Bacterial loads in the lung (a) and spleen (b) of BALB/c mice intravenously infected with ~1 × 10^5^ CFU *M. tuberculosis* H37Rv, Δ*casT*, Δ*cas6* or Δ*csm5* at the times indicated post-infection. Error bars: SEM. Group size: n = 4. (b) (a) Necroscopy of the lung and spleen of BALB/c mice infected with ~1 × 10^5^ H37Rv, Δ*casT*, Δ*cas6* or Δ*csm5* at 56 and 160 d post-infection. (b) Spleen weight of mice at 160 d post-infection. (c) Pathology of the lung in BALB/c mice infected with wild-type *M. tuberculosis* (H37Rv), Δ*casT*, Δ*cas6* or Δ*csm5*. H&E stained mouse lung tissue (50 × magnification, scale bar = 300 μm) at 14 d, and 160 d post-infection. Inset images (1000 × magnification, scale bar = 20 μm) show *M. tuberculosis* bacilli. Experiments in A were performed at least three times. Images presented in B and care from representative experiments

MTB secreted proteins play important roles in the process of MTB infection [20]. To evaluate any potential role of selected CRISPR/Cas proteins in MTB pathogenesis *in vivo*, we tested the pathogenicity of CRISPR/Cas knock-out strains in a BALB/c mouse MTB challenge model. 1 × 10^5^ CFUs [21] of wild-type (H37Rv), Δ*casT* (all CRISPR/Cas proteins knocked out), Δ*cas6* (Cas6 functions independently of the intracellular effector complex and is secreted), or Δ*csm5* (present in the intracellular effector complex and is secreted) strains were injected intravenously (tail vein) in 6–8 week old BALB/c mice (n = 4 per group) and bacterial numbers in the lung and spleen were monitored by CFU counts over a period of 160 days (complemented strains were not included due to practical constraints) (Supplementary Table 3; Figure 2; Supplementary Figure 2). Lung CFU counts for the Δ*casT*, Δ*cas6* and Δ*csm5* strains at 160 days were significantly lower than for the wild-type (Wild-type: 1.79E+06 ± 9.46E+05; Δ*casT*: 4.82E+03 ± 2.08E+03; Δ*cas6*: 7.0E+03 ± 1.6E+03; Δ*csm5*: 4.10E+04 ± 9.45E+03. P < 0.001, two-tailed unpaired *t*-test), with Δ*casT* and Δ*cas6* strain colony counts being significantly lower than those for Δ*csm5* (P = 0.0022 and P = 0.0112, respectively). A similar pattern was observed in spleen CFU counts (Figure 2(a), Supplementary Table 3). Less extensive tissue damage was observed during lung necroscopy of BALB/c mice infected with these strains relative to the wild-type, and similar reductions in the size and weight of the spleen were recorded Figure 2(b).

Consistent with the above findings, we observed differences in HE-stained sections of tissues from wild-type mice and those infected with CRISPR/Cas protein deletion strains Figure 2(c). Mice infected with any of the CRISPR/Cas protein deletion strains showed less severe interstitial pneumonia and inflammation throughout the lung than in mice infected with the wild-type H37Rv strain. Infiltration of inflammatory cells into the alveolar walls was also less marked, and alveolar air spaces were not obliterated to the same extent.

The above results suggest that CRISPR/Cas proteins may play roles in MTB pathogenicity *in vivo*, the pathogenicity of Cas6 being more pronounced than that of Csm5.

### MTBCas6 induces macrophage and lung fibroblast apoptosis

**Figure 3.**
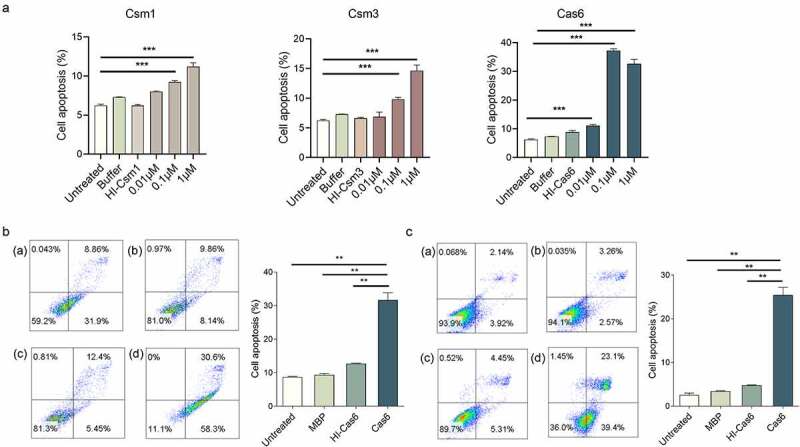
MTBCas6 induces macrophage and lung fibroblast apoptosis. (a) Dose–response curves of Cas6, Csm1 and Csm3 induction of THP-1 macrophage apoptosis. PMA-differentiated THP-1 cells were incubated with a range of concentrations of 6X His tagged- Csm1, Csm3 and Cas6, buffer, or 1 μM Heat-inactivated 6X His tagged-Csm1 (HI-Csm1), Csm3 (HI-Csm3) or Cas6 (HI-Cas6) (boiled for 1 h before addition to cells as a control for specificity) for 24 h. Apoptosis was detected by Annexin V/PI staining. (b-c) Raw264.7 cells (b) and L929 cells (c) were treated with 0.1 µM Cas6 for 24 h and apoptosis was detected by Annexin V/PI staining (a. untreated b. MBP protein c. Heat-inactivated MTBCas6 protein (HI-Cas6) d. MTBCas6 protein). Data presented are means ± SD from representative experiments with three independent biological replicates. **P < 0.01, Student’s *t*-test

Secreted MTB proteins associated with virulence are reported to be potent inducers of cell-mediated host immune processes, such as cell apoptosis, which are crucial in the course of MTB infection [22]. To determine if MTB CRISPR/Cas proteins can induce apoptosis, we incubated phorbol 12-myristate 13-acetate (PMA) differentiated human THP-1 macrophage cells with different concentrations of recombinant secreted proteins Cas6, and Csm1, Csm2, Csm3, Csm5, and Csm6. Cas6, Csm1 and Csm3, but not Csm2, Csm5 or Csm6-induced macrophage apoptosis, Cas6 inducing the strongest response (Figure 3(a), Supplementary Figure 3). Levels of Cas6-induced apoptosis increased at concentrations from as low as 0.01 µM up to the maximum concentration tested (1 µM), implying that secreted CRISPR/Cas proteins may induce apoptosis in a dose-dependent manner. When we repeated this apoptosis assay for Cas6 using mouse RAW264.7 macrophages and mouse L929 lung fibroblasts cells, similar phenomena were observed Figure 3(b,c). Apoptosis, however, was not detected in BHK21 baby hamster kidney cells or DC2.4 mouse dendritic cells (Supplementary Figure 4 A-B). These data suggest that Cas6 plays an important role in TB-related immune processes.

### MTBCas6 induces apoptosis via interactions with the cell surface

**Figure 4.**
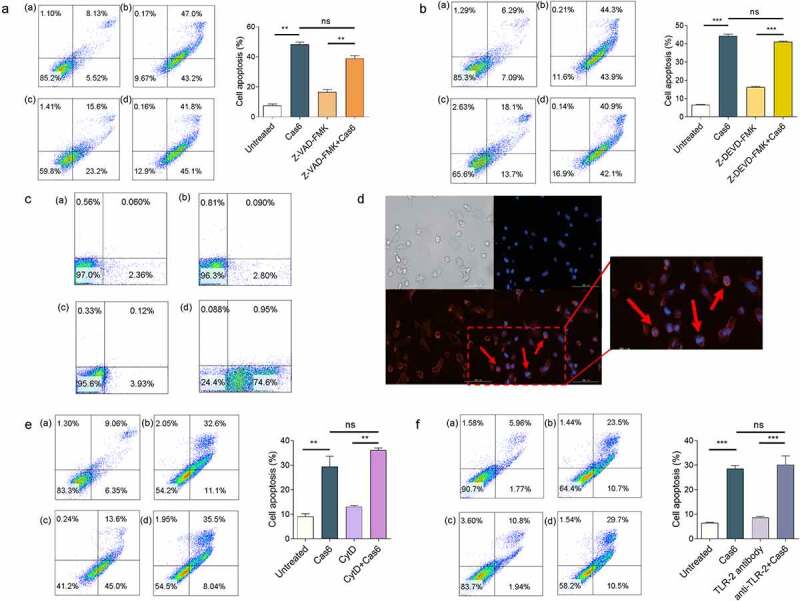
MTBCas6-induced apoptosis involves interactions with the cell surface. (a-b) MTBCas6-induced apoptosis is not a classical caspase-dependent pathway. Apoptosis was determined by Annexin V/PI double staining flow cytometry. (a) Apoptosis in THP-1 cells treated with Z-VAD-FMK (10 μM) then incubated with MTBCas6 (0.1 μM) for 24 h (a. untreated b. MTBCas6 protein c. Z-VAD-FMK d. Z-VAD-FMK + MTBCas6 protein). (b) THP-1 cells were incubated with Z-DEVD-FMK (20 μM), then treated with MTBCas6 (0.1 μM) for 24 h (a. untreated b. MtbCas6 protein c. Z-DEVD-FMK d. Z-DEVD-FMK + MTBCas6 protein). (c-d) MTBCas6 is mainly located at the surface of THP-1 cells. (c) THP-1 cells incubated with anti-histidine were stained with Alexa Fluor–conjugated secondary antibody and analyzed on a FACS Calibur (a. untreated b. his antibody with Alex 647 c. MTBCas6 d. MTBCas6 + his antibody with Alex 647). (d) THP-1 cells treated with histidine-tagged MTBCas6, stained with anti-histidine followed by Alexa Fluor 647-conjugated secondary antibody (red) and DAPI (blue) were viewed with a Cytation^TM^ 3 reader. Bar = 100 µm. (e) MTBCas6 induces apoptosis via direct interactions with a cell surface protein. THP-1 cells were pretreated with CytD (10 μM) and apoptosis was induced by incubation with MTBCas6 protein (0.1 μM) (a. untreated b. MTBCas6 protein c. CytD d. CytD + MTBCas6 protein). (f) MTBCas6 induction of apoptosis does not occur via Toll-Like Receptor-2 (TLR-2). THP-1 cells were first incubated with TLR-2 antibody (5 µg/ml) for 2 h, then treated with MTBCas6 (0.1 μM) for 24 h (a. untreated b. MTBCas6 protein c. TLR-2 antibody d. TLR-2 antibody with MTBCas6 protein). Data presented are the mean ± SD of representative experiments with three independent biological replicates, **P < 0.01, ***P < 0.001, ns = non-significant, student’s *t*-test

To explore the mechanism of MTBCas6-induced apoptosis, THP-1 cells were incubated with the broad-spectrum caspase inhibitor, ZVAD-FMK, or Z-DEVD-FMK, a specific caspase3 inhibitor. Apoptosis signals were not affected, suggesting that Cas6-induced apoptosis is not a classical caspase-dependent pathway Figure 4(a,b). We next investigated if MTBCas6-induced apoptosis is associated with a surface receptor. Flow cytometry and immunofluorescence microscopy of THP-1 cells incubated first with His-tagged MTBCas6 for 10 min and then with an anti-His antibody followed by an Alexa Fluor–conjugated secondary antibody showed that MTBCas6 is indeed mainly located at the surface of THP-1 cells Figure 4(c,d). In addition, pretreatment of cells with Cytochalasin D (CytD), an actin depolymerization drug (to inhibit phagocytosis) did not decrease MTBCas6-induced apoptosis, suggesting that MTBCas6 induces apoptosis via interactions with a cell surface protein Figure 4(e). We then examined if, like secreted virulence factors LpqH, a 19-kDa MTB glycolipoprotein (p19), and ESAT-6, MTBCas6 induces macrophage apoptosis via Toll-Like Receptor-2 (TLR-2). Levels of apoptosis, however, were not reduced in TLR-2 antibody-pretreated THP-1 cells treated with MTBCas6 Figure 4(f), suggesting that MTBCas6-induced apoptosis may be TLR-2-independent.

### MTBCas6 induces the NF-κB signaling pathway

**Figure 5.**
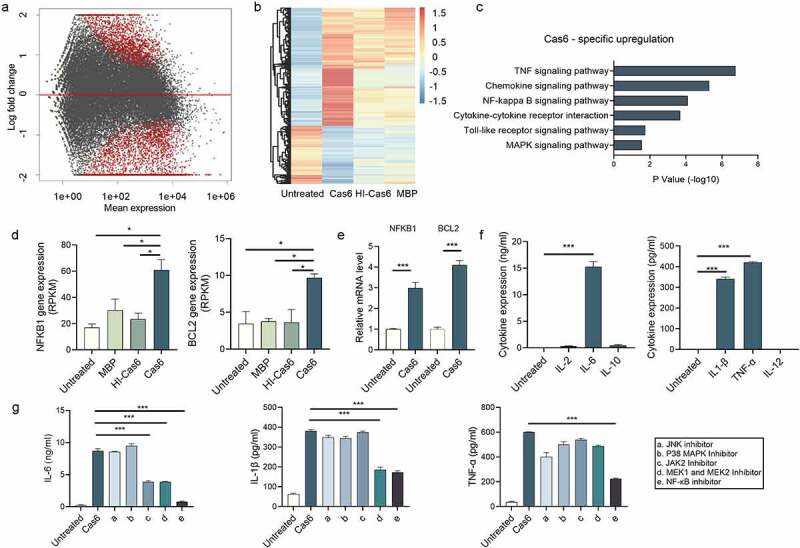
MTBCas6-induced release of IL-6, IL-1β and TNF-α from THP-1 cells is NF-κB-dependent. (a-b) THP-1 cells were treated with or without MTBCas6 (1 µg/ml), heat-inactivated MTBCas6 (HI-Cas6) (1 µg/ml) or MBP (1 µg/ml) for 24 h and total RNA was extracted and sequenced on an illumina HiSeqXten System. (a) MA-plot and (b) heat map of the 851 up-regulated and 767 down-regulated genes specific to the transcriptome of Cas6-THP-1 cells. (c) KEGG and GO gene functional annotation of MTBCas6-induced differentially expressed genes. (d) RNA-seq data for two highly differentially expressed genes associated with the NF-κB pathway. (e) Quantitative PCR analysis of NFKB1 and BCL2 expression in THP-1 cells treated with MTBCas6. (f) Levels of IL-1α, IL-1β, IL-6, IL-12, IL-2, IL-4, IL-10 and TNF-α in the supernatants of THP-1 cells treated with MTBCas6 (1 µg/ml) for 6 h as determined using ELISA assays. (g) IL-6, IL-1β, and TNF-α levels in supernatants from THP-1 cells stimulated with MTBCas6 (1 µg/ml) for 6 h together with NF-κB inhibitor CELASTROL (10 μM), JNK inhibitor SP600125 (10 μM), P38 MAPK Inhibitor SB203590 (10 μM), JAK2 Inhibitor AG490 (20 μM) or MEK1 and MEK2 Inhibitor PD98059 (10 μM). Data presented are means ± SD from representative experiments with three independent biological replicates, *P < 0.05, ***P < 0.001, student’s *t*-test

To gain insight into which signaling pathway(s) are induced by MTBCas6 in macrophages, we compared the transcriptomes of THP-1 cells pre- and post-stimulation with MTBCas6, obtained using RNA-seq. 1974 genes were upregulated in MTBCas6-stimulated THP-1 cells (Cas6-THP-1) compared to unstimulated THP-1 cells (WT-THP-1), and 969 genes were down-regulated (cutoff parameters: fold-change ≥2; *p* < 0.05) Figure 5(a,b). After upregulated and downregulated genes whose expression also varied when THP-1 cells were stimulated with heat-inactivated MTBCas6 (HI-Cas6-THP-1) or MBP (MBP-THP-1) (951 and 1077 up-regulated and 157 and 140 down-regulated genes, respectively (p value < 0.05)), were excluded, 851 up-regulated and 767 down-regulated genes were found to vary specifically in Cas6-THP-1 cells.

KEGG enrichment analysis of the 851 genes up-regulated specifically in Cas6-THP-1 cells indicated that the TNF and NF-κB signaling pathways were enriched. Gene Ontology (GO) analysis of these genes also pointed to the involvement of inflammatory response and immune response biological processes Figure 5(c).

Transcription factor NF-κB signaling pathways are reported to regulate innate immunity. Inflammatory cytokines, such as IL-6, IL-1β and TNF-α, are involved in activating cells of the immune system in response to mycobacterial infection [23,24]. Genes associated with the NF-κB pathway, were among the most strongly upregulated genes in Cas6-THP-1 cells (Figure 5(c), Supplementary Figure 5). Validation of the upregulation of NF-κB pathway associated genes NFκB1, an important protein in the NF-kappa-B complex, and BCL2, a protein regulated by NF-κB signaling pathway [25], in THP-1 cells pre- and post- MTBCas6 stimulation using qPCR gave results consistent with our findings in the transcriptome analysis Figure 5(d,e). These data provide evidence suggesting that MTBCas6 stimulation of THP-1 cells leads to marked activation of the NF-κB pathway at the transcriptional level.

We then investigated if MTBCas6 stimulation of THP-1 cells leads to the release of TB-associated pro-inflammatory cytokines such as the interleukins IL-1β, IL-2, IL-6, IL-10, and IL-12, and TNF-α Figure 5(f). IL-1β, IL-6, and TNF-α release were detected, levels of IL-6 release being the highest. We then incubated MTBCas6 with THP-1 cells pretreated with inhibitors specific to different cell signaling pathways to try to identify which signaling pathway leads to this MTBCas6-promoted cytokine secretion. Inhibition of NF-κB with CELASTROL markedly decreased MTBCas6-induced IL-6, IL-1β, and TNF-α expression, while inhibiting JAK2, MEK1 or MEK2 decreased the expression of these cytokines to a lower degree, and inhibiting JNK or p38 MAPK did not markedly affect their expression Figure 5(g). These results suggest that the NF-κB signaling pathway may be the primary signaling pathway for MTBCas6-induced release of TB-associated cytokines IL-1β, IL-6, and TNF-α.

## Discussion

The canonical involvement of CRISPR/Cas proteins in prokaryotic CRISPR defense systems against invading nucleic acids has been studied extensively. Non-canonical roles in pathogenesis involving intracellular targeting of certain virulence-associated endogenous mRNAs have also been reported for some Type I and Type II CRISPR/Cas system proteins, but no such roles have yet been established for Type III system proteins. Here, we have shown that certain MTB CRISPR/Cas proteins (Type III-A system) are secreted antigens that induce host immune responses. Cas6, in particular, provoked IFN-γ release from PBMCs from active TB patients, its deletion attenuated virulence in a murine MTB challenge model, and it induced apoptosis of macrophages and lung fibroblasts. We found that MTBCas6 interacts with the surface of cells in a caspase and TLR-2 independent manner, and that stimulation of THP-1 macrophages with MTBCas6 led to upregulated expression of genes associated with the NF-κB pathway and to higher levels of cytokines known to activate immune system cells in response to MTB infection. Our findings suggest that secreted MTB CRISPR/Cas proteins can trigger cellular immune responses associated with virulence.

MTB’s pathogenic mechanisms are markedly different from other bacteria. Rather than exerting virulence via toxin production, MTB’s virulence factors play an important role in its pathogenicity; it is the interplay between pathogen virulence factors and host tissue cell components that determine if disease will develop. While on the one hand the host immune system produces large numbers of macrophages to control MTB infection, on the other hand, MTB deploys specialized secretory systems to deliver virulence proteins that modulate a variety of host cellular pathways enabling MTB to evade macrophage killing [26]. MTB secreted proteins thus play an important role in virulence, and the study of secreted proteins has been important for identifying virulence factors and antigens that might serve as biomarkers of infection [27]. Several MTB secreted proteins have previously been shown to contribute to macrophage apoptosis. ESAT-6, for instance, inhibits the activation of ASK1 (apoptosis signal-regulating kinase 1) via TLR-2, TLR-7 and TLR-9 ligands [28], and LpqH, a 19 kDa MTB lipoprotein, induces macrophage apoptosis via the TLR-2 and TLR-4 pathways [9]. While MTB CRISPR/Cas proteins have appeared on lists of proteins present in culture filtrates in some proteomic studies [12,13], their secretion has not previously been either validated or investigated. Here, we found that MTB CRISPR/Cas proteins Csm1, Csm3, Csm5 and Csm6, and Cas6 are secreted (Figure 1) and induce a variety of cellular immune responses, including the induction of T-cell responses (Csm1, Csm6, Cas6; Figure 1), macrophage apoptosis (Csm1, Csm3, Cas6; Figure 3), and activation of the NF-κΒ signaling pathway (Cas6; Figure 5). Cas6, in particular, appears to make an important contribution to MTB virulence.

Cas6 is an endoribonuclease that typically participates in intracellular CRISPR/Cas defense against invading nucleic acids through its involvement in generating mature crRNAs by site-specific cleavage within repeat sequences in repeat-spacer CRISPR arrays [29]. Here, our animal study suggested that its non-canonical role as a virulence factor may be more important than that of other CRISPR/Cas proteins; the pathology of mice infected with the Δ*cas6* strain was similar to that of mice infected with a strain in which all the CRISPR/Cas proteins were deleted (Δ*casT*) and was less severe than that induced in mice infected with a Δ*csm5* strain Figure 2(a). TB pathogenesis relies on an interplay between the host defense system and the different survival strategies employed by MTB via expression of various virulence factors. Here, MTBCas6 triggered cell apoptosis and induced a host immune response. Cell apoptosis is an innate immune response against certain pathogens. However, intracellular pathogens such as MTB disable cells such as macrophages to establish an infection, and then replicate within infected macrophages and subsequently infect surrounding macrophages.

Apoptosis is a complex process, involving many components and complex signaling pathways. NF-κB is known to regulate downstream cytokines, inflammatory molecules, and proteins associated with apoptosis [30]. Here, MTBCas6 stimulation of macrophages activated the macrophage NF-κΒ signaling pathway, provoking release of interleukins IL-6, and IL-1β and TNF-α, cytokines important for the activation of immune system cells [31–34], suggesting that secreted MTBCas6 can modulate major immune signaling pathways and promote the expression of certain inflammatory cytokines. Other MTB-secreted proteins also modulate immune signaling pathways; PtpA and Rv1808, for example, manipulate cytokine profiles via co-activation of the MAPK and NF-κB signaling pathways [23,35]. Our results are also consistent with previous findings indicating that MTB-infected macrophages produce high levels of pro-inflammatory cytokines (TNF-α, IL-1β, IL-12, and IL-6) in mouse lung tissues in response to MTB infection [36], and that TNF-α is required for controlling acute MTB infection; in TNF-α or 55-kDa TNF receptor deficient mice, MTB infection results in rapid death, with substantially higher bacterial burdens compared to control mice [37,38]. IL-6 is also known to be important in the initial innate response to MTB infection; IL-6 induces mycobacterial growth inhibition in macrophages and its deficiency impairs IFN-γ release [39]. NF-κB is considered indispensable in the control of pro-inflammatory expression and has been implicated in the pathogenesis of a number of inflammatory diseases, including MTB infection [40,41] where it regulates the production of cytokines by MTB-infected macrophages [42].

Our study has some limitations. While we have confirmed the presence of CRISPR/Cas proteins in MTB culture filtrates and provided convincing data on the biological significance of these proteins, we did not investigate the mechanism by which they are secreted. It will be important to determine which of the four major MTB secretion systems is involved in CRISPR/Cas protein secretion. In addition, while we were able to determine that these proteins remain at the cell surface and function in a caspase and TLR-2 independent manner, we were unable to identify the specific cell surface receptor these proteins interact with.

Accumulating evidence suggests that CRISPR/Cas systems may have broader functions in bacterial physiology [43], and may target their own genes, altering virulence and evading mammalian host immunity. *Pseudomonas aeruginosa* CRISPR/Cas system (Type I-F) alters virulence, for example, by degrading the mRNA of the quorum-sensing (QS) master regulator LasR, and *Francisella novicida* (Type II) Cas9 utilizes small CRISPR/Cas-associated RNAs (scaRNAs) to repress an endogenous bacterial lipoprotein transcript that triggers a proinflammatory innate immune response [18,44], thus dampening the host response and promoting virulence. To date, reports of these non-canonical functions have been limited to Type I and Type II systems, and reported functions have all been carried out intracellularly. This is the first report of such a non-canonical role for Type III system proteins and the first report that CRISPR/Cas proteins are secreted as virulence factors.

In recent years, CRISPR ribonucleoprotein effector systems have become indispensable tools in biological research, enabling facile and efficient targeted gene editing in a wide range of applications, including knocking-out genes [45], regulation of endogenous gene expression [46], live-cell labeling of chromosomal loci [47] and high-throughput gene screening [48]. Gene editing technologies using CRISPR/Cas proteins also have potential clinical applications [49,50]. For example, cells engineered to have enhanced proliferation and to boost effector gene programs in response to T cell receptor stimulation may hold promise as cancer immunotherapies [51]. Our findings may have implications for the development of CRISPR-based gene editing therapies. It will be important to determine if the CRISPR/Cas protein induced immune responses observed here are a widespread feature of CRISPR/Cas systems in general. If so, any host immune responses induced by CRISPR/Cas proteins employed in gene-editing applications should be carefully evaluated and ameliorated during the development of CRISPR-based gene-editing therapies.

This study has shown that MTB CRISPR/Cas proteins are secreted and has provided strong experimental evidence of non-canonical functions of these proteins, in particular Cas6, in virulence. Our findings suggest that MTB CRISPR/Cas proteins not only act as a defensive shield against invading nucleic acids, but also as a sword of secreted virulence factors and immunogens that pierce host defense systems and induce various immune responses. These proteins may have clinical applications as biomarkers for MTB infection or in the development of TB vaccines.

## Materials and methods

### Bacterial strains and growth conditions

Experiments were performed with the *M. tuberculosis* reference strain H37Rv, and its isogenic mutants Δ*cas6* and Δ*cas6*(*pcas6*). Isogenic mutants Δ*casT* (deletion of all CRISPR/Cas proteins), Δ*cas6*, Δ*csm5* and complemented strain Δ*cas6*(*pcas6*) were constructed according to a previously published method [52]. *M. tuberculosis* strains were grown in Middlebrook 7H9 medium supplemented with 0.5% glycerol, 10% oleic acid-albumin-dextrose-catalase (OADC) and 0.05% Tween-80.

### Isolation of secreted proteins from M. tuberculosis H37Rv

*M. tuberculosis* H37Rv was grown in Sauton’s medium containing 0.5% Tween-80 for 30 d. The culture supernatant was filtered three times with a 0.2 μm filter and the culture filtrate was concentrated using 3 KD ultrafiltration tubes. Western blotting was performed with individual anti-Csm1-6/Cas6 sera. GroEL (an unsecreted protein) was used as a negative control, and ESAT-6 and Ag85b (secreted proteins) were used as positive controls (Abcam, Cambridge, MA). Total protein from *M. tuberculosis* H37Rv cell extracts were used as *in vivo* controls.

### Clinical sample collection and IFN-γ ELISPOT assays

Blood samples from 15 healthy individuals and 85 patients confirmed clinically to have active TB (demographic information given in Supplementary Table 2) were obtained at Beijing Chest Hospital, Capital Medical University, Beijing. Clinical sample collection was approved by the Beijing Chest Hospital Ethics Committee. IFN-γ release ELISpot assays were performed using T-SPOT.TB (Oxford Immunotec, UK), according to the manufacturer’s instructions. Briefly, PBMCs were collected by Ficoll-Paque density gradient centrifugation of 5 ml heparinized blood samples and 2.5 × 10^5^ cells per well were seeded into 96-well plates. Cells were incubated with either MTBCsm6, MTBCas6, or MTBCsm1 proteins (200 pmol) or ESAT6/CFP10 (50 μl), medium or Phytohemagglutinin (PHA) (as negative and positive controls, respectively) at 37°C. Secreted IFN-γ was captured by specific antibodies on the PVDF membrane on the well surface. Spots formed as a result of this reaction spot forming cells (SFCs) were counted. Positive responses were recorded when the test protein cell had ≥6 spots, negative control cells had >10 SFUs and PHA control. Positive responses were recorded when the test protein cell had ≥6 spots, negative control cells had >10 SFUs and PHA control wells had ≥40 SFUs.

### Transcriptome generation, functional annotation, and classification

Total RNA was prepared from three biological replicates using TRIzol (Invitrogen, Carlsbad, CA, USA) according to the manufacturer’s instructions, resuspended in RNase-free water and stored at −80°C. mRNA was fragmented and converted into a strand-specific RNA-seq library using the KAPA Stranded RNA-Seq kit (KAPA Biosystems, USA) according to the manufacturer’s instructions. For both cDNA and genomic libraries, cluster generation was performed on an Illumina C-bot (Illumina, San Diego, CA, USA) and 2 × 150 bp paired-end sequencing was performed using an Illumina HiSeq X Ten (Illumina, San Diego, CA, USA). The quality of raw sequencing data was checked by fastQC (https://www.bioinformatics.babraham.ac.uk/projects/fastqc). Low-quality reads were filtered using Trimmomatic-0.36 [53], and STAR [54] was used to map filtered reads to the human reference genome (GRCh38). Duplicate reads were filtered using Picard-tools-2.1.0 [55]. Differentially expressed genes were determined using the DEseq package [56] in R, with cutoff parameters set at fold-change: 2; *p* < 0.05. Figures were plotted using ggplot2 (https://ggplot2.tidyverse.org). Gene Ontology (GO) analysis (https://david.ncifcrf.gov/tools.jsp) was performed to obtain functional classifications and KEGG pathway analysis (http://www.genome.jp/kegg) was performed to determine the involvement of differentially expressed genes in different biological pathways.

### qRT-PCR analysis

Total RNA was extracted from three independent cultures of THP-1 cells grown in RPMI 1640 (Gibco, Paisley, UK) supplemented with 10% fetal bovine serum (Gibco, Paisley, UK) using TRIzol (Invitrogen, Carlsbad, CA, USA) according to the manufacturer’s instructions. Complementary DNA (cDNA) was synthesized using the SuperScript III First-Strand Synthesis System (Invitrogen), according to the manufacturer’s instructions. Quantitative PCR was performed using SYBR Select Master Mix (Thermo Fisher Scientific) on a QuantStudio7 (Applied Biosystems). All reactions were performed in a total reaction volume of 10 μl, with initial denaturation at 95°C for 2 min, followed by 40 PCR cycles consisting of annealing at 55°C for 15 s and extension at 72°C for 30 s, and final denaturation at 95°C for 30 s. Gene expression levels were normalized to β-actin. The following primers were used: β-actin, forward primer: 5ʹ-CTTCCAGACTCCTGAGGACCAA, reverse primer: 5ʹ-ACATAGCCAGCACGCTCAGCAA; NFKB1, forward primer: 5ʹ-AACAGAGAGGATTTCGTTTCCG, reverse primer: 5ʹ-TTTGACCTGAGGGTAAGACTTCT; BCL-2, forward primer: 5ʹ-GGTGGGGTCATGTGTGTGG, reverse primer: 5ʹ-CGGTTCAGGTACTCAGTCATCC.

### Antibody production and Western blotting

Specific antibodies against purified recombinant MTB Csm1-6 and Cas6 proteins were raised at the Institute of Genetics and Development Biology, Chinese Academy of Sciences, by immunizing 6–8 week old BALB/c mice on four occasions (days 1, 22, 36 and 50) with the corresponding protein. Mice were sacrificed after a further 10 days and serum was collected and stored at −80°C until required. Samples for analysis by Western blotting were subjected to 10% SDS-PAGE before transferring to a nitrocellulose membrane. The nitrocellulose membrane was blocked for 1 h at room temperature in 1× PBST with 5% (w/v) nonfat milk and then incubated with a 1:1000 dilution of the appropriate antiserum overnight at 4°C. After washing the membrane three times with PBST, bound primary antibody was detected by incubating with a secondary HRP-linked rabbit anti-mouse IgG (1:5000, GE Healthcare) and chemiluminescent substrate (ECL-plus substrate, GE Healthcare) according to the manufacturer’s instructions.

## Apoptosis assays

PMA-differentiated L929, and RAW264.7 cell lines (China Infrastructure of Cell Line Resources, Beijing, China) and THP-1 cell lines (American Type Culture Collection (ATCC), Manassas VA, USA) were seeded, respectively, in 12-well flat-bottomed tissue culture plates at a density of 3–5 × 10^5^ cells per well and allowed to adhere or differentiate at 37°C. Cells were washed and medium was replaced 3 h before any treatment. Cells were incubated for 2 h with His-tagged Cas6 at a range of concentrations from 0.01 µM to 1 µM. After washing, cells were incubated at 37°C, 5% CO_2_ for up to 24 h. Culture supernatants and cells were collected and washed with cold PBS. Apoptosis was measured by Annexin V and PI staining. Cells were collected and washed with cold PBS and resuspended in Annexin V-FITC binding buffer (10 mM HEPES, pH 7.4, 140 mM NaCl, 2.5 mM CaCl_2_). An aliquot of 100 µl was removed and mixed with 5 µl of Annexin V-FITC. The mixture was vortexed and incubated for 5 min at room temperature in the dark. Cells were then washed once with binding buffer, and the volume was increased to 500 µl with binding buffer mixed with 5 µl PI for analysis by flow cytometry. As Cas6 was purified from *E. coli*, we controlled for the possibility of nonspecific effects of lipopolysaccharides by including treatment groups incubated with heat-inactivated Cas6 alone. Native Cas6 was used to directly compare its effects in these assays.

## Expression and purification of recombinant Cas6

The *M. tuberculosis* Cas6 (Rv2824c) gene was amplified by PCR from genomic DNA and cloned into a modified pET28a plasmid that included an MBP-tag. *Escherichia coli* BL21(DE3)/Rv2824c cells were grown to OD600 = 0.6. Protein expression was induced overnight at 16°C with 0.4 mM Isopropyl β-D-1-thiogalactopyranoside (IPTG, Sigma, St. Louis, MO, USA). Cells were subsequently pelleted, resuspended in 50 mM Tris-HCl (pH 7.5), 500 mM NaCl and 0.5 mM phenylmethylsulfonyl fluoride (PMSF), and disrupted by sonication. After centrifugation at 15,000 rpm for 40 min at 4°C, the supernatant was loaded on an Ni-NTA agarose bead (GE Healthcare) column equilibrated with 50 mM Tris-HCl, pH 8.0, containing 400 mm NaCl and glycerin 5% v ⁄ v (buffer A). After washing the column with 100 mM imidazole in buffer A, bound Rv2824c was eluted with 500 mM imidazole in buffer A.

## Cell culture and microscopy

For fluorescence microscopy, PMA-differentiated THP-1 cells were seeded in 12-well flat-bottomed tissue culture plates at a density of 3–5 × 10^5^ cells per well and allowed to adhere or differentiate at 37°C. THP-1 cells treated for 10 min with histidine-tagged Cas6 were fixed with paraformaldehyde, stained with anti-histidine followed by Alexa Fluor 647–conjugated secondary antibody and then viewed with a Cytation^TM^ 3 reader. To analyze the binding of Cas6 to the cell surface, treated cells were incubated with anti-histidine, stained with Alexa Fluor–conjugated secondary antibody and then analyzed on a FACS Calibur equipped with Cell Quest software (Becton Dickinson).

## Detection of cytokines by ELISA

IL-1α, IL-1β, IL-2, IL-4, IL-6, IL-10, IL-12, and TNF-α concentrations in supernatants from THP-1 cells treated with 1 µg/ml Cas6 were measured using commercially available sandwich ELISA kits (Biolegend). All procedures were performed according to the manufacturer’s instructions. Results were analyzed by one-way ANOVA. P values <0.05 were considered as statistically significant.

## Statistical analysis

Statistical analysis was performed using GraphPad Prism software (GraphPad Software Inc., San Diego, CA, USA). Data are expressed as the mean ± SD. All P-values were calculated by two-tailed unpaired t test. P < 0.05 was considered statistically significant (*P < 0.05, **P < 0.01 and ***P < 0.001).

## Supplementary Material

Supplemental Material

## Data Availability

Raw sequence data have been deposited in the Genome Sequence Archive [57] of the National Genomics Data Center [58], China National Center for Bioinformation/Beijing Institute of Genomics, Chinese Academy of Sciences under accession number HRA001361, and are publicly accessible at https://ngdc.cncb.ac.cn/gsa-human.
