# Supplementary material for: M. tuberculosis CRISPR/Cas proteins are secreted virulence factors that trigger cellular immune responses
Source: Virulence. 2021 Dec 9;12(1):3032–44. doi: 10.1080/21505594.2021.2007621 (PMC8667911; doi:10.1080/21505594.2021.2007621)
Supplement: Supplemental Material [file KVIR_A_2007621_SM4971.pdf]

Supplementary materials

for

***M. tuberculosis* CRISPR/Cas proteins are secreted virulence factors that trigger cellular immune responses**

Jianjian Jiao[a,d], Nan Zheng[a,d], Wenjing Wei[a,d*], Joy Fleming[a], Xingyun Wang[a*], Zihui Li[b], Lili Zhang[a*], Yi Liu[b], Zongde Zhang[b], Adong Shen[c], Chuanyou Li[b,#], Lijun Bi[a,#] & Hongtai Zhang[a,#]

[a] Key Laboratory of RNA Biology and State Key Laboratory of Biomacromolecules, CAS Center of Excellence in Biomacromolecules, Institute of Biophysics, Chinese Academy of Sciences, Beijing 100101, China

[b] Beijing Chest Hospital, Capital Medical University; Beijing Tuberculosis and Thoracic Tumor Research Institute; Beijing Key Laboratory for Drug Resistant Tuberculosis Research, Beijing 101149, China.

[c] Beijing Pediatric Research Institute, Beijing Children's Hospital, Capital Medical University, 100045, Beijing

[d] University of Chinese Academy of Sciences, Beijing 100049, China

Jianjian Jiao, Nan Zheng and Wenjing Wei contributed equally to this work.

Supplementary Tables

Table S1. Secreted CRISPR/Cas proteins

Table S2. Antigenicity of *M. tuberculosis* CRISPR proteins Csm1, Csm6 and Cas6 as determined by antigen-specific IFN-γ release from PBMC cells evaluated using T-SPOT.TB (ELISPOT) assays

Table S3. Bacterial loads in the lung or spleen of BALB/c mice infected with H37Rv, Δ*casT*, Δ*cas6* or Δ*csm5* from 2 to 160 days

Supplementary Figures

Fig. S1. Cas6 protein is secreted

Fig. S2. Construction of Δ*casT*, Δ*cas6* and Δ*csm5* mutant strains of *M. tuberculosis* H37Rv

Fig. S3. Csm2, Csm5 and Csm6 do not induce apoptosis of THP-1 cells

Fig. S4. Cas6 does not induce apoptosis of DC2.4 cells or BHK21 cells

Fig. S5. Transcriptome data for genes associated with NF-κB signaling pathway in THP-1 cells stimulated with MTBCas6

Table S1. Secreted CRISPR/Cas proteins

Protein	Reported to be secreted in proteomic studies	Experimentally verified here to be secreted
Cas6	6 peptides[a]	Yes
Csm1	10 peptides[a]	Yes
Csm2	No[a]	Yes
Csm3	8 peptides[a,b]	Yes
Csm4	No[a]	No
Csm5	3 peptides[a]	Yes
Csm6	19 peptides[a,b,]	Yes

[a]Kelkar et al., 2011, [b] Tucci et al., 2020

References

a. Kelkar DS, Kumar D, Kumar P, et al. Proteogenomic analysis of Mycobacterium tuberculosis by high resolution mass spectrometry. Mol Cell Proteomics. 2011 Dec;10(12):M111.011627.

b. Tucci P, Portela M, Chetto CR, González-Sapienza G, Marín M. Integrative proteomic and glycoproteomic profiling of Mycobacterium tuberculosis culture filtrate. PLoS One. 2020 Mar 3;15(3):e0221837.

Table S2. Antigenicity of *M. tuberculosis* CRISPR proteins Csm1, Csm6 and Cas6 as determined by antigen-specific IFN-γ release from PBMC cells evaluated using T-SPOT.TB (ELISPOT) assays

	Active TB (n = 85)		HC (n = 15)	
Antigen	Positive[1]	Sensitivity (%)[2]	Positive[1]	Sensitivity (%)[3]
Csm1	41	48	6	40
Csm6	51	60	5	33.3
Cas6	36	42	3	20
ESAT-6	61	72	4	26.7
CFP-10	49	58	3	20

[1] Positive was defined as SFUs ≥6 in 2.5×10^5 cells

[2] Percentage of responding patients out of all TB patients tested.

[3] Percentage of responding healthy donors out of all healthy donors tested.

Raw data:

Active TB Patient	-ve Control	Antigen A	Antigen B	+ve Control	Csm6	Csm1	Cas6
1	0	5	2	904	0	0	0
2	0	21	126	712	7	15	1
3	0	4	38	611	2	1	2
4	0	43	0	702	8	13	7
5	0	9	30	769	13	9	0
6	0	14	41	799	8	14	3
7	1	14	0	978	2	3	1
8	0	0	3	600	1	1	1
9	0	66	20	670	72	46	65
10	0	12	10	694	19	16	10
11	0	33	5	679	4	7	6
12	2	26	11	999	2	3	0
13	0	2	1	530	1	0	0
14	0	4	12	281	0	2	0
15	0	0	0	476	1	0	0
16	0	158	11	332	8	2	2
17	0	0	0	263	7	10	15
18	0	91	55	75	1	1	1
19	0	10	2	427	8	16	5
20	0	12	4	142	2	5	3
21	0	6	4	545	3	1	2
22	0	44	19	97	24	15	3
23	0	4	24	577	5	7	12
24	0	0	0	889	6	4	8
25	1	20	59	988	0	1	1
26	0	1	0	1070	5	8	7
27	0	487	20	640	5	1	3
28	0	117	26	909	56	69	28
29	0	2	1	1116	8	5	10
30	0	55	287	1126	16	8	15
31	0	163	47	795	1	4	3
32	0	139	41	848	15	18	15

33	0	9	0	623	6	5	7
34	0	14	6	989	18	16	19
35	0	1	0	778	8	10	8
36	1	56	72	807	23	25	14
37	0	113	42	406	9	12	21
38	1	194	17	1080	1	5	4
39	0	28	93	759	18	23	22
40	0	5	4	1036	39	53	51
41	0	49	0	1063	4	2	1
42	0	6	4	915	6	3	8
43	1	129	29	1086	4	6	1
44	0	11	16	639	10	7	9
45	0	6	15	461	3	2	5
46	0	5	76	893	6	5	2
47	0	84	12	507	2	1	0
48	0	5	14	1312	26	17	35
49	0	64	50	1118	6	2	5
50	0	4	0	697	16	23	13
51	1	1	5	1089	6	0	7
52	0	23	8	1120	34	3	1
53	0	10	3	958	30	22	14
54	0	52	224	908	4	6	2
55	0	10	32	544	1	2	6
56	1	215	550	740	6	5	9
57	0	38	6	1118	0	0	2
58	0	28	101	748	0	1	2
59	0	866	243	1113	8	5	2
60	0	4	2	969	2	1	2
61	0	4	1	996	1	3	2
62	0	7	0	1027	2	5	1
63	0	18	177	1049	17	24	30
64	1	14	10	697	1	4	1
65	0	147	627	902	40	51	98
66	0	12	322	850	2	3	7
67	0	1	1	1084	237	16	5
68	0	7	103	274	31	24	17
69	0	14	5	76	1	0	6
70	0	123	1	306	14	8	1
71	0	23	1	325	9	13	15
72	0	84	6	690	11	7	2
73	0	3	5	816	5	7	10
74	0	56	1	303	10	3	3
75	0	41	196	754	2	5	3
76	1	1	1	709	6	13	9

77	0	11	1	785	7	3	2
78	0	84	86	447	6	5	1
79	0	8	7	500	8	11	4
80	0	55	22	591	16	12	9
81	0	36	236	653	10	15	5
82	0	22	47	582	23	24	2
83	0	0	0	515	4	6	1
84	0	31	35	335	6	5	4
85	0	0	0	672	8	3	1

**Healthy donor**	**-ve Control**	**Antigen A**	**Antigen B**	**+ve Control**	**Csm6**	**Csm1**	**Cas6**
1	0	0	1	405	5	2	4
2	0	0	0	672	2	0	1
3	0	0	0	1092	11	16	3
4	0	0	0	772	4	7	3
5	0	1	0	32	4	1	2
6	0	0	0	515	35	1	0
7	0	45	20	252	4	7	3
8	0	3	58	334	0	1	0
9	0	29	12	736	4	3	1
10	1	0	0	952	5	3	3
11	0	25	4	1238	11	9	11
12	0	2	0	958	2	0	2
13	0	2	2	937	0	2	2
14	1	11	2	1040	6	10	6
15	1	0	0	1122	16	9	11

- : Negative control
+: PHA (phytohaemagglutinin) control
A: ESAT-6 antigen
B: CFP10 antigen
Test wells were defined as positive if they contained ≥6 spots and at least twice as many spots as the negative control. PHA control wells were required to have at least 50 SFUs (spot-forming units), and negative control wells to have fewer than 10 SFUs.

Table S3. Bacterial loads in the lung or spleen of BALB/c mice infected with H37Rv, Δ*casT*, Δ*cas6* or Δ*csm5* from 2 to 160 days.

**Lung (cfu)**	**Time (days)**	**H37Rv**	**Δ*casT***	**Δ*csm5***	**Δ*cas6***
	2	1.94E+03 ±3.40E+02	1.32E+03 ±3.07E+02	6.20E+03 ±8.33E+02	2.75E+03 ±3.42E+02
	14	3.68E+05 ± 6.06E+04	1.28E+04 ±2.42E+03	5.53E+04 ±2.41E+04	1.26E+04 ±5.29E+03
	28	1.64E+06 ±5.80E+05	1.25E+04±5.47E+03	8.63E+04 ±4.83E+04	1.46E+04 ±5.58E+03
	42	2.11E+06 ±5.52E+05	4.88E+04 ±2.02E+04	3.47E+05 ±1.15E+05	1.30E+05 ±3.50E+04
	56	1.46E+06 ±5.09E+05	2.23E+04 ±1.25E+04	9.35E+04 ±4.88E+04	4.90E+04 ±9.59E+03
	84	6.81E+05 ±1.18E+05	3.15E+03 ±6.19E+02	4.55E+04 ±1.36E+04	5.70E+03 ±9.59E+03
	160	1.79E+06 ±9.46E+05	7.00E+03 ±2.08E+03	4.10E+04 ±9.45E+03	4.82E+03 ±1.36E+03
**Spleen (cfu)**	**Time (days)**	**H37Rv**	**Δ*casT***	**Δ*csm5***	**Δ*cas6***
	2	2.03E+04 ±1.49E+03	1.37E+04 ±3.90E+03	6.05E+04 ±9.57E+03	2.80E+04 ±4.23E+03
	14	2.37E+06 ±5.36E+05	6.30E+04 ±7.07E+03	2.45E+05 ±4.20E+04	6.43E+04 ±4.91E+03
	28	2.51E+06 ±1.30E+06	4.35E+04 ±1.94E+04	2.05E+05 ±8.16E+04	3.60E+04 ±1.75E+04
	42	2.00E+06 ±6.53E+05	3.95E+04 ±1.84E+04	3.50E+05 ±1.16E+05	4.05E+04 ±1.26E+04
	56	1.70E+05±4.48E+04	2.80E+04 ±7.55E+03	1.70E+05 ±3.79E+04	3.60E+04 ±2.10E+04
	84	1.92E+05±5.96E+04	6.60E+02 ±7.66E+01	9.29E+04 ±1.50E+04	1.19E+03 ±1.54E+02
	160	3.63E+05 ±9.71E+04	1.33E+03 ±9.45E+01	4.08E+04 ±2.92E+04	2.34E+03 ±1.19E+03

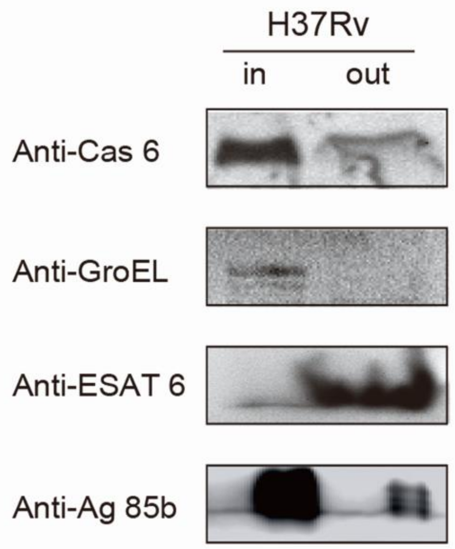

Fig. S1 Cas6 protein is secreted. Western blotting of cellular extracts and culture filtrates of MTB H37Rv probed with anti-Cas6 sera (1 μg/ml). Controls: GroEL (unsecreted), ESAT-6 (secreted via the ESX-1 system), Ag 85b (secreted via the TAT pathway). 50 μg of all protein samples were analyzed.

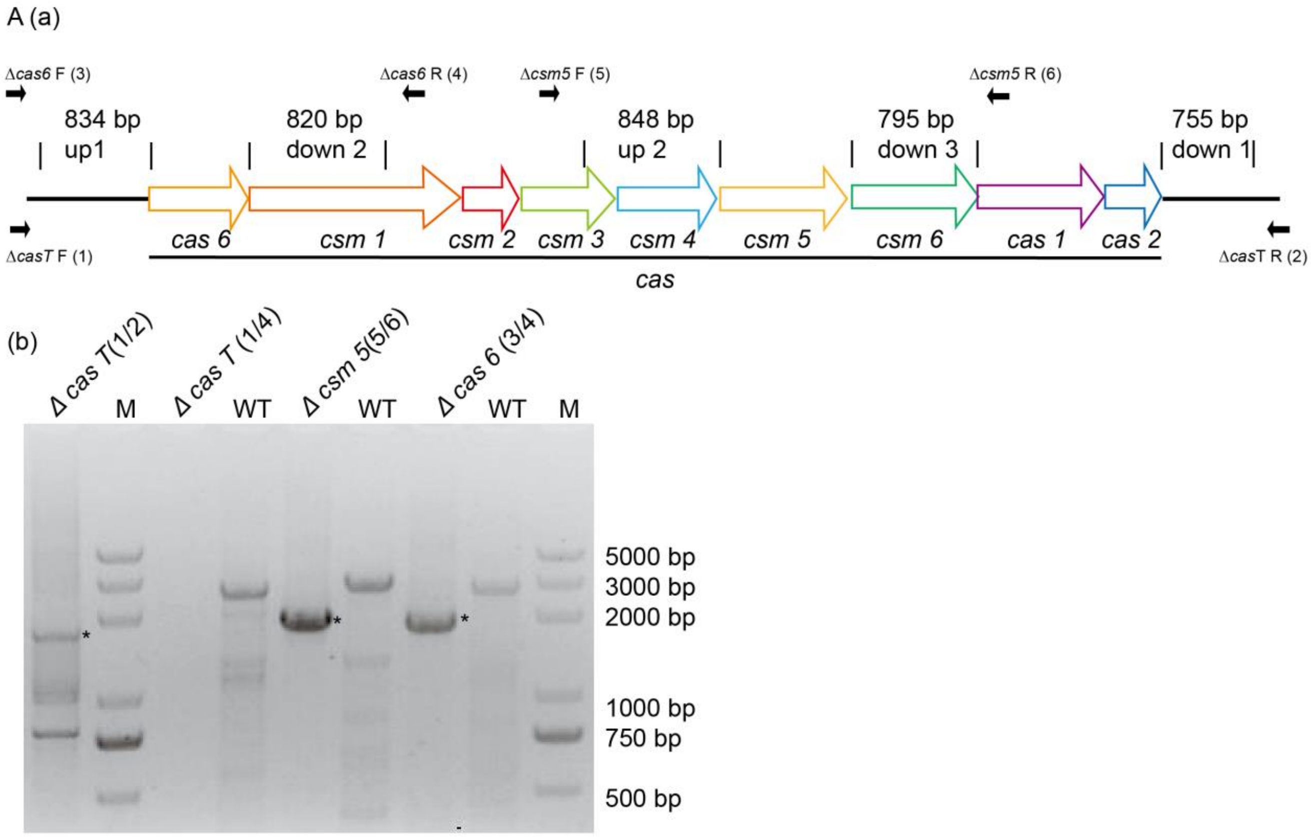

Fig. S2 Construction of Δ*casT*, Δ*cas6* and Δ*csm5* mutant strains of *M. tuberculosis* H37Rv. Δ*casT*, Δ*cas6* and Δ*csm5* mutants were constructed via homologous recombination with p1NIL-PGOAL, resulting in the deletion of the corresponding gene coding region (A). (B) Mutants were confirmed by PCR. The presence of amplicons from the wild type strain was used as a negative control. Primers used: Δ*csm5*, forward primer: 5'-AGCAGTGCGAGCCAGTCCT, reverse primer: CGTGGCTACGGGCAGGTC; Δ*cas6*, forward primer: CCGTGTTGGGCAGCAGCAG, reverse primer: ATGAGAGCAGTTGGGCACG; Δ*casT*, forward primer: GGAAGTCAACTAGAGCGGGTGT, reverse primer: CACGAGTACTGTCATCACTATTGG.

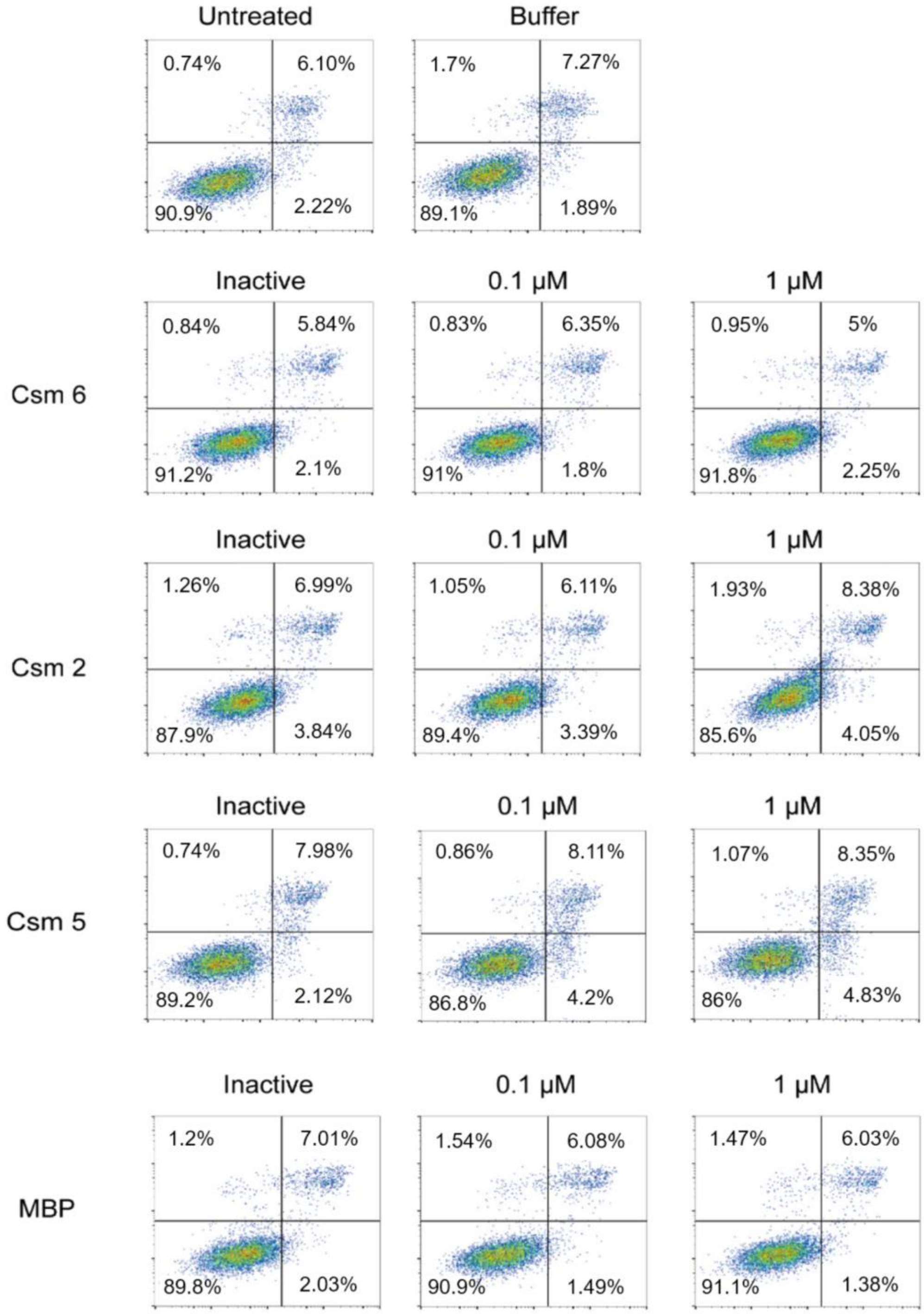

Fig. S3 Csm2, Csm5 and Csm6 do not induce apoptosis of THP-1 cells. PMA-differentiated THP-1 cells were incubated with a range of concentrations of 6X His tagged- Csm2, Csm5 and Csm6, buffer, or 1 μM inactivated 6X His tagged-Csm2, Csm5 or Csm6 (boiled for 1 h before addition to cells as a control for specificity) for 24 h. Apoptosis was analyzed by flow cytometry (annexin-V/propidium iodide method). Cells were regarded as apoptotic when they were tagged by annexin-V-FITC (X-axis) and excluded propidium iodide (Y-axis). Experiments were performed at least three times.

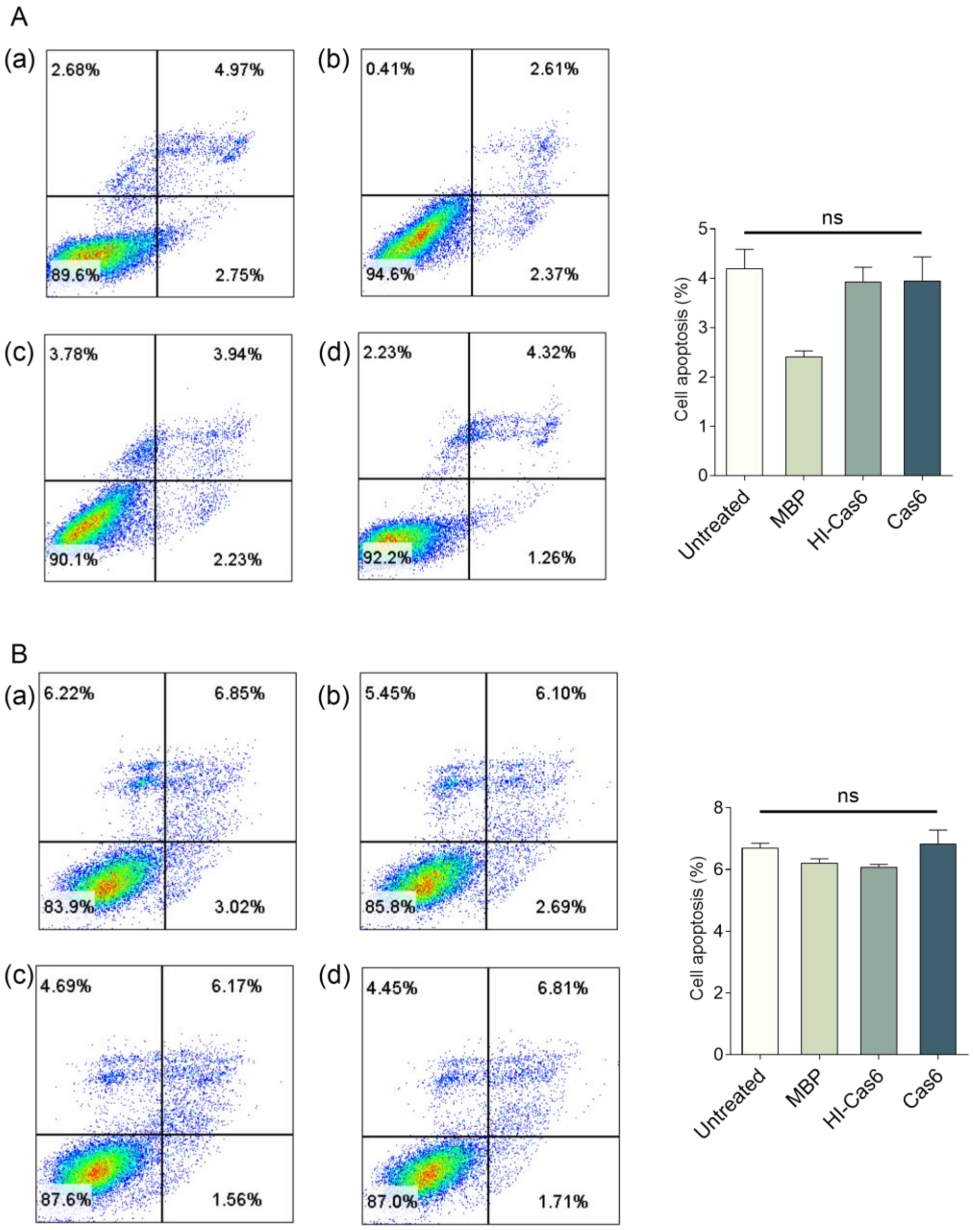

Fig. S4 Cas6 does not induce apoptosis of DC2.4 cells or BHK21 cells. DC2.4 cells (A) and BHK21 cells (B) were treated with 0.1 μM Cas6 for 24 h. Apoptosis was detected by Annexin V/PI staining and flow cytometry. a. untreated b. MBP protein c. Heat-inactivated MTBCas6 protein (HI-Cas6) d. MTBCas6 protein. Data presented are means ± SD from representative experiments with three independent biological replicates.

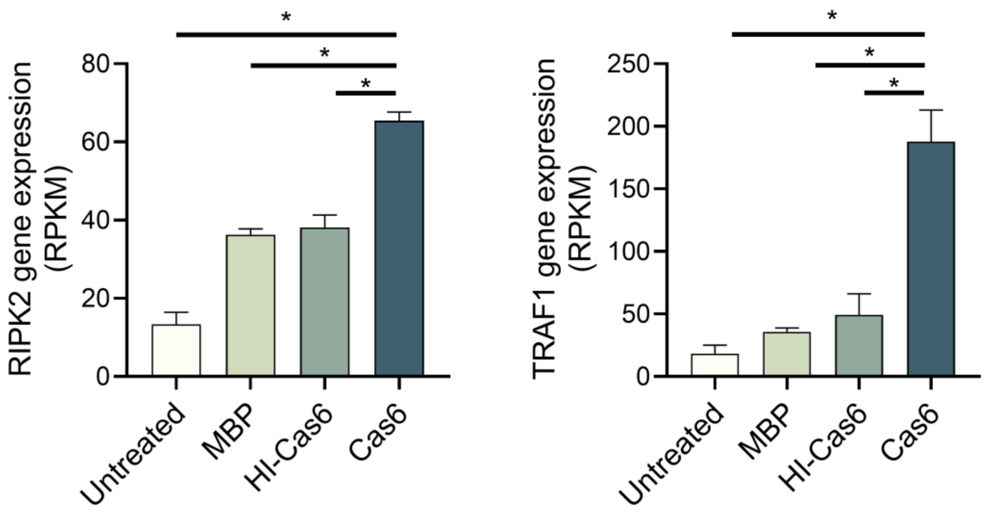

Fig. S5 Transcriptome data for genes associated with NF-κB signaling pathway in THP-1 cells stimulated with MTBCas6. RNA-Seq data for RIPK2 and TRAF1differentially-expressed genes associated with the NF-κB pathway. THP-1 cells were treated with or without MTBCas6 (1 μg/ml), heat-inactivated MTBCas6 (1 μg/ml) or MBP (1 μg/ml) for 24 h and total RNA was extracted and sequenced on an Illumina HiSeqXten System. Data presented are means ± SD with three independent biological replicates, *P < 0.05, Student's t-test.
